# Effectiveness and safety of acupuncture in the treatment of chronic severe functional constipation: A protocol for systematic review and meta-analysis

**DOI:** 10.1097/MD.0000000000024589

**Published:** 2021-02-19

**Authors:** Daocheng Zhu, Jinyu Hu, Zhenhai Chi, Xilin Ouyang, Wei Xu, Zhaona Luo, Chao Cheng, Jiajia Wu, Rixin Chen, Lin Jiao

**Affiliations:** a The Affiliated Hospital of Jiangxi University of Traditional Chinese Medicine; b College of Acupuncture-Moxibustion and Tuina, Jiangxi University of Traditional Chinese Medicine, Nanchang, China.

**Keywords:** acupuncture, chronic severe functional constipation, protocol, systematic review

## Abstract

**Background:**

Acupuncture has been widely used clinically to relieve chronic severe constipation. However, the efficacy of acupuncture in the treatment of chronic severe constipation is uncertain. The purpose of this study is to determine the effectiveness and safety of acupuncture in the treatment of chronic severe constipation.

**Methods:**

Search PubMed, Cochrane Library, Embase, China National Knowledge Infrastructure Database, Wanfang Database, China Science, and Technology Journal Database, China Biomedical Literature Database, and search related randomized controlled trials. Two reviewers will independently select studies, collect data, and evaluate methodological quality through the Cochrane Deviation Risk Tool. Revman V.5.3 will be used for meta-analysis.

**Results:**

This study will evaluate the current status of acupuncture treatment for chronic severe constipation, aiming to illustrate the effectiveness and safety of acupuncture treatment.

**Conclusion:**

This study will provide a basis for judging whether acupotomy is effective in treating chronic severe constipation.

**INPLASY registration number:**

INPLASY202070002

## 1 Introduction

Chronic constipation is a common gastrointestinal disease. the main clinical manifestations are infrequent bowel movement, excessive straining, stiff stool, and/or incomplete evacuation sensation.^[1]^ patients with severe chronic constipation have complete spontaneous bowel movement (CSBMs) not more than twice a week. stool is hard, often tense and feels incompletely evacuated.^[2]^ According to epidemiological studies found that the global prevalence of chronic idiopathic constipation is about 16%, affecting about 17.1% of the European population,^[3]^ 12% to 19% of the North American population,^[4]^ and 10.8% of the Asian population.^[5]^ Furthermore, studies have reported that the prevalence of constipation in women is more than twice that of male^[6,7]^ of the same age. Woman with constipation also often have lower urinary tract symptoms^[8]^ bladder hyperactivity and urinary incontinence. It is seriously affect the quality of life and health,^[9]^ constipation can also lead to loss of work and productivity, psychological, and psychiatric symptoms.^[10]^

The actual etiology of Chronic constipation remains unknown, but evidence suggests it is usually caused by many factors such as diet, medications, metabolic or neurological disorders, and psycho social issues, as well as dysfunction of colonic motility and the defecation process.^[11]^ At present, the routine treatment for chronic constipation mainly includes stimulants and osmotic drugs, such as laxatives and stimulant drugs, but it is easy to repeat after withdrawal, and the efficacy and safety of drugs are not guaranteed. Almost 50% of patients with chronic constipation are dissatisfied with the treatment of these drugs.^[12]^ A clinical trial in which daily administration of 1 to 2 mg of procapride (agonist of serotonin receptor 4) was reported to restore BMS to normal in 37.9% of patients with severe chronic constipation.^[13]^ However, the adverse cardiac effects caused by some prokinetic agents cannot be ignored.^[14]^

Acupuncture has a long history in treating obstinate constipation, Emerging evidence suggests that acupuncture may increase weekly CSBMs, decrease constipation symptoms, and improve quality of life in patients with chronic constipation.^[15]^ However, studies assessing whether patients can benefit from acupuncture remain scarce.^[16]^

The focus of this study is the efficacy of acupuncture therapy for Chronic Severe Functional Constipation related clinical symptoms. Therefore, we conducted this study to systematically evaluate the impact of acupuncture on Chronic Severe Functional Constipation. It can provide a basis for the diagnosis and treatment of acupuncture therapy Chronic Severe Functional Constipation.

## 2 Methods

### 2.1 Criteria for inclusion

#### 2.1.1 Type of studies

All randomized controlled trials with acupuncture treatment of chronic intractable constipation will not be restricted by publication status or written language. Semi-randomized controlled trials and animal studies, conference abstracts will be excluded.

#### 2.1.2 Type of participant

All cases included in the trial involved participants who had been diagnosed with chronic functional constipation. Diagnosis was based on Rome III criteria for chronic functional constipation and severe constipation was defined as 2 or fewer CSBMs per week with hard stools, frequent straining, and the sensation of incomplete evacuation.^[17]^ A CSBM was defined as a bowel movement with a sensation of complete evacuation that occurred without use of any medication or other methods to assist defecation in the previous 24 hours. There is no restriction of age, sex, or race limit.

#### 2.1.3 Type of intervention

##### 2.1.3.1 Experimental interventions

The interventions of the experimental group will include any type of clinical acupuncture for chronic functional constipation alone to improve the symptoms. include Acupotomy, needle therapy, Electroacupuncture, fire needle, etc acupuncture Combined with other interventions such as massage, moxibustion, herbal medicine, qigong, functional exercise, and other mixed therapies will be excluded.

##### 2.1.3.2 Control interventions

The controlled intervention accepts any international recognized therapy, such as traditional medicine, moxibustion, etc. Non-intervention and Placebo will also be included. Studies comparing the therapeutic effects of different types of acupuncture manipulations will be excluded.

#### 2.1.4 Types of outcome measurements

##### 2.1.4.1 Primary outcome

The changes from baseline in mean CSBMs per week, mean SBMs per week, mean stool consistency, and mean straining scores and PAC-QOL score will be adopted as the primary outcomes.

##### 2.1.4.2 Secondary outcomes

The safety assessment will be considered a secondary outcome.

### 2.2 Search methods for identification of studies

#### 2.2.1 Electronic data sources

Search from the establishment of the database to December 1, 2020. We will search 4 English medical electronic databases including PubMed, EMBASE, Cochrane Central Register of Controlled Trials, Web of Science. Chinese literature will be searched through China's 4 major databases, including Chinese Biomedical Literature Database, Wanfang Database, the Chongqing VIP and Chinese National Knowledge Infrastructure. Search terms consist of disease (Chronic Severe Functional Constipation, Constipation, Dyschezia, Colonic Inertia) and intervention (acupuncture, needle, Electroacupuncture and research types (randomized controlled trial, controlled clinical trial, random trials). The searching strategy of PubMed is presented in Table 1.

**Table 1:** Search strategy used in PubMed database.

Number	Search items
#1	randomized controlled trial [pt]
#2	controlled clinical trial [pt]
#3	randomized [tiab]
#4	clinical trials as topic [mesh: noexp]
#5	randomly [tiab]
#6	trial [ti]
#7	or/ #1–#7
#8	animals [mh] not humans [mh]
#9	#7 not #8
#10	Constipation [MeSH]
#11	Chronic constipation [All Fields)
#12	Colonic inertia [all fields)
#13	Gastrointestinal motility [all fields)
#14	Colonic motility [all fields)
#15	Intestinal dysmotility [all fields)
#16	Functional colonic diseases [all fields)
#17	Dyschezia [all fields)
#18	Or/#10–#17
#19	Newborn or child or baby or babies or infant or youth or pediatric or paediatric or toddler or preschool or pre-school[all fields)
#20	#18 and #19
#21	Acupuncture Theraphy [MeSH]
#22	Acupunture [all fields)
#23	Acupotomy [all fields)
#24	Needle therapy [all fields)
#25	Pinprick [all fields)
#26	Electroacupuncture [all fields)
#27	Auricular acupuncture [all fields)
#28	Catgut embedding [all fields)
#29	Or/ #21–#28
#30	#9 and #20 and #29

#### 2.2.2 Search other resources

We will search the Clinicaltrials.gov, China Clinical Trial Registry, and the relevant conference papers related to Massage for treatment of congenital muscular torticollis, proposing to obtain unpublished or ongoing has not uploaded trial data.

### 2.3 Data collection

#### 2.3.1 Selection of studies

**Figure 1:**
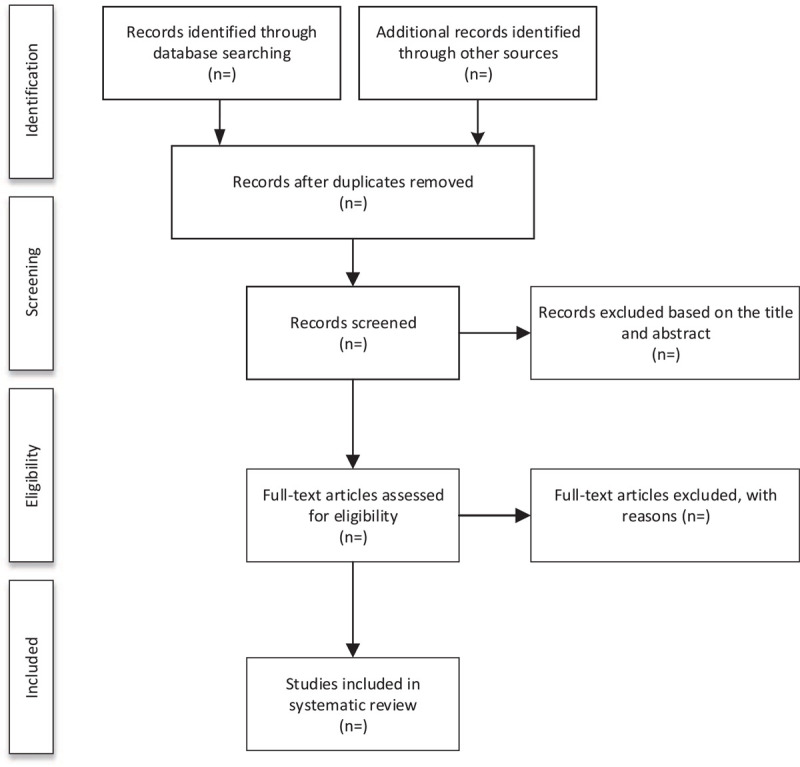
Flow diagram of study selection process.

We will export all the studies of electronic searches according to the search strategy into the EndNote software (V.X9) for management. Literatures obtained from other sources will also be imported into EndNote and remove the duplicate dates. Then, 2 researchers (DC and ZH) will independently screen the research literature that met the Eligibility criteria by reading the titles and abstracts. Irrelevant literature will be deleted. If they cannot determine whether can be included in the study by the title and abstract of the literature, the Full-text articles will be screened for judgment. After this step, the 2 researchers (DC and ZH) conducted a cross-check. During this process, if there appear any disagreement, it will be resolved through group discussions or decided by the third-party reviewer (JY). The specific process and results of studies selection will be shown in the flow chart of Figure 1.

#### 2.3.2 Data extraction and management

The research team will determine the standard data extraction form in advance, and then the 2 researchers (DC and ZH) will independently extract the following information from the included studies into the form:

1. Study basic information: year of publication, source and ID, the first author name, publication language and country, article title of the study, etc.
2. Participant information: sex, age, country, ethnicity, diagnostic criteria, basic disease information, sample size, etc.
3. Trial characteristic: number of groups, test design method, random control method, blind method, result analysis method, Number of participants in observation, and control groups, etc.
4. Interventions and Controls: the method of intervention, the name of the operation method, the duration and frequency of treatment, and the treatment cycle, name and type of the control, combination therapy, etc.
5. Outcomes: various evaluation standards and types, primary and secondary outcomes (change in clinical symptom score, evaluation of imaging changes), timeline for assessment, length of follow- up period, adverse event, etc.

After extracting the data, the 2 researchers (DC and ZH) will conduct a cross-check. If there is any ambiguity, it will be discussed and decided by all members of the research group. The extracted data will be listed in the standard data table (Microsoft excel 2016), and JY will check it to ensure the data is accurate.

### 2.4 Assessment of risk of bias in included studies

Two reviewers (JY and DC) of the team will use the risk of bias assessment tool by the Cochrane Collaboration to independently evaluate the quality of the final included trials.^[18]^ The risk assessment indicators will include the following 7 contents: random sequence generation, allocation concealment, blinding of participants and personnel, blinding of outcome assessment, incomplete outcome data, selective reporting, and other sources of bias. Each domain of the study will be judged as high-risk, low-risk, and unclear risk of bias as the evaluation results. Our reviewers (JY and DC) will strictly check the assessment results in accordance with the evaluation rules. If there is any ambiguity and disagreement, we will be resolved through consultation. In addition, it can also be judged by the third reviewer (XL).

### 2.5 Data synthesis

#### 2.5.1 Measures of treatment effect

We will designate 2 reviewers (DC and ZH) use Review Manager Software (RevMan 5.3) and Stata software to conduct statistical analysis and synthesize all data. For categorical data, we will use the risk ratio and 95% confidence intervals (95%CIs) to calculate and summarize data. For continuous data, Mean difference and 95% CI will be used to present the data synthesis outcome. If the outcome variables of different measurement scales are measured, standardized mean difference analysis with 95%CI will be performed.

#### 2.5.2 Management of missing data

If data is missing in the included study, we will contact the author by email or phone to obtain the original data and incomplete data. If the contact fails, We will follow the Cochrane Handbook methods and estimate the missing means and standard deviations of the baseline change based on existing baseline data and other data.^[19]^

#### 2.5.3 Assessment of heterogeneity

χ^2^ test in forest plot and I^2^ statistic will be used to assess the heterogeneity. When performing a Chi-squared test, a *P* value less than .10 will be considered significant.^[20]^ If performing I^2^ statistic verification, the effect of heterogeneity on the Meta-analysis will be quantified by calculating the I^2^ value, The specific I^2^ value follows the measurement standard as follows: if the I^2^ statistic is ≤50%, the research results might be considered no heterogeneity, and The fixed- effects model will be used for data synthesis and analysis; if the I^2^ statistic is 50% ≤ I^2^≤ 90%, means extensive heterogeneity; when 75%≤I^2^≤100% will be considered as important heterogeneity and A random effects model will be applied, and sensitivity analysis is used to assess the impact of all included trials on the final outcome results.

#### 2.5.4 Assessment of reporting biases

For the inclusion of more than ten trials, a funnel plot will be drawn to detect the reporting bias. The publication bias for binary and the asymmetry of funnel plot will be assessed quantitatively using the Egger test.

#### 2.5.5 Subgroup analysis

If there are significant heterogeneities in the included studies, the STATA software will be used for subgroup analysis and meta-regression analysis according to the characteristics of the test subjects, sample size, different massage intervention methods, quality of included trials, etc.

#### 2.5.6 Sensitivity analysis

We will evaluate the robustness of the meta-analysis results through sensitivity analysis, and exclude such as small-sample trials and low-quality trials to explore the impact of trial quality on efficacy estimates. In addition, we will conduct a second meta-analysis based on the results of the sensitivity analysis, summarize in tables and discuss.

#### 2.5.7 Grading the quality of evidence

Two reviewers will independently evaluate the quality of the evidence of all research outcomes by using the Grading of Recommendations Assessment, Development, and Evaluation system. And according to the Grading of Recommendations Assessment, Development, and Evaluation rating standards, use “high”, “moderate”, “low”, “very low” 4 levels to rate the quality of evidence.^[21,22]^

#### 2.5.8 Ethics and dissemination

The second study based on the literature in this study does not involve the personal data of the research trials, so it does not require ethical approval. We will provide systematic evaluation and evidence by evaluating the treatment of TCM by accupuncture, and provide methods and ideas for the clinic. The systematic review and result will be published in a peer-reviewed journal.

## 3 Discussion

Acupuncture has been widely used in the treatment of chronic severe functional constipation. A high-quality randomized controlled trial clinical study shows that acupuncture can effectively relieve the symptoms of chronic constipation.^[23,24]^ In addition, related basic experiments have proved that acupuncture can help regulate reflex, control gastrointestinal motility and secretion by regulating serotonin receptors, thereby promoting gastrointestinal peristalsis.^[25]^ But the clinical efficacy of acupuncture has not been scientifically and systematically evaluated. This study aims to evaluate the clinical efficacy and safety of acupuncture in the treatment of chronic severe functional constipation. The conclusions of this study can provide evidence-based medicine recommendations for acupuncture treatment of chronic severe functional constipation.

Research limitations: First of all, in the process of acupuncture and moxibustion treatment, the choice of treatment, the choice of treatment site, time and frequency may be heterogeneous. Second, this study has set strict inclusion criteria, and the inclusion of high-quality literature may have less impact. The reliability of systematic review depends to a large extent on comprehensiveness and methodological quality. Third, the included studies do not limit language types, and there are certain language biases.

## Author contributions

**Data curation:** Daocheng Zhu, Zhenhai Chi.

**Formal analysis:** Daocheng Zhu, Zhenhai Chi.

**Investigation:** Daocheng Zhu, Zhenhai Chi.

**Methodology:** Daocheng Zhu, Fuqiang Yuan.

**Project administration:** Lin Jiao.

**Software:** Daocheng Zhu, Zhenhai Chi.

**Supervision:** Lin Jiao.

**Validation:** Lin Jiao.

**Visualization:** Daocheng Zhu,

**Writing – original draft:** Daocheng Zhu, Zhenhai Chi.

**Writing – review & editing:** Lin Jiao.
